# Proceedings of the 22^nd^ Academic Conference of the Bauru School of Dentistry “Dr. Waldyr Antonio Janson”, the 16^th^ Academic Conference of Speech-Language Pathology and Audiology of the Bauru School of Dentistry “Dr. Kátia de Freitas Alvarenga” and the 3^rd^ Meeting of Latin American Region of the IADR and 8^th^ Meeting of the Venezuelan Division of the IADR

**DOI:** 10.1590/S1678-77572009000700001

**Published:** 2009

Dear Readers,

In this special issue of 2009, the Journal of Applied Oral Science (JAOS) is dedicated to publishing an extremely reach and valuable material. We are publishing full papers, awarded with special prizes, and abstracts of different areas of knowledge of two events held in our campus of the University of São Paulo in Bauru, namely the 22^nd^ Academic Conference of the Bauru School of Dentistry “Dr. Waldyr Antonio Janson” (May, 13-16) and the 16^th^ Academic Conference of Speech-Language Pathology and Audiology of the Bauru School of Dentistry “Dr. Kátia de Freitas Alvarenga” (August, 26-29). Additionally, we are publishing the full papers derived from the excellent presentations during the Symposium “Two decades of ART: success through research”, which occurred during the 3^rd^ Meeting of Latin American Region of the IADR and 8^th^ Meeting of the Venezuelan Division of the IADR (Isla Margarita, Venezuela, November, 11-14).

It is important to clarify that all the material published in this special issue went through the rigorous peer-review evaluation process and language editing established in the journal rules. We are extremely grateful to all the referees involved in this time-consuming activity.

Finally, we take this opportunity to congratulate the organizers of the three outstanding scientific events aforementioned. We understand how difficult this task is! We hope this initiative of the JAOS helps to spread all the good science and clinical work presented by all the authors who attended such meetings.

With kind regards,

